# Supplementary material for: Functional Analysis of Zebrafish socs4a: Impacts on the Notochord and Sensory Function
Source: Brain Sci. 2022 Feb 10;12(2):241. doi: 10.3390/brainsci12020241 (PMC8869963; doi:10.3390/brainsci12020241)

**Figure S1. Expression analysis of *socs4a*.** Whole-mount *in situ* hybridization analysis of *socs4a* on embryos at 0 hpf (A, B), 24 hpf (C, D), 31 hpf (E, F) and 3 dpf (G, H) using either sense (S) or anti-sense (AS) probes, as indicated. The embryos in panels A and B are upright and imaged in laterally, with all other embryos positioned with their anterior to the left and imaged either laterally (C, D, G, H) or dorsally (E, F). The images in panels C and D are identical to those used in Fig. 1 F and G to aid comparison.

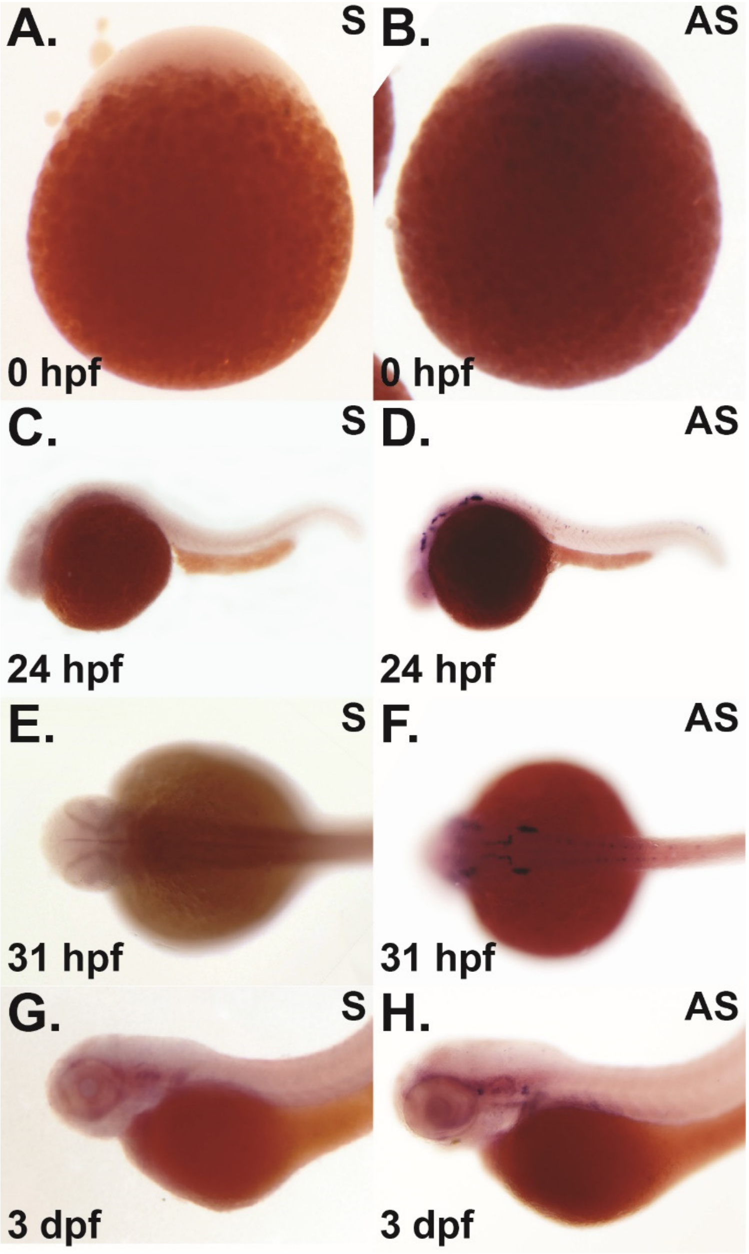

**Figure S2. Confirmation of efficacy of *socs4a* ATG morpholino.** Analysis of *in vitro* transcription and translation products of *socs4a* mRNA in the presence of control (Ctl) or ATG MO (ATG) or with no mRNA template (NT) as a control. Size markers are shown, along with an arrow indicating the relative position of the Socs4a protein around ~44 kDa, the asterisk showing a non-specific protein product, and the box highlighting the region of this gel displayed in Fig. 2B.

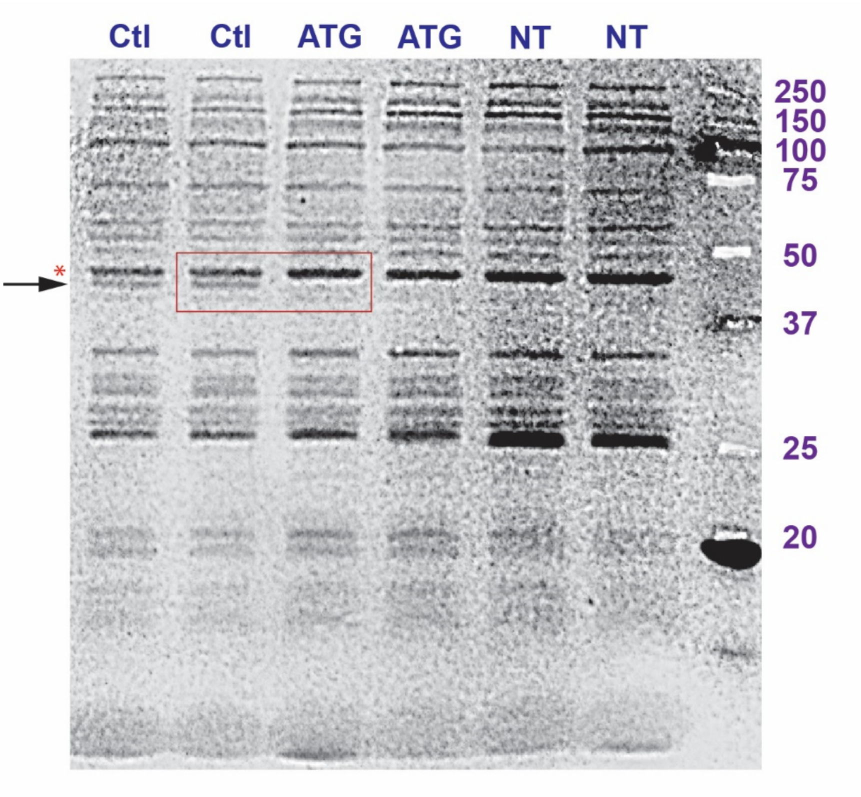

Supplement: Supplementary file 1 [file brainsci-12-00241-s001.zip › brainsci-1466099-supplementary.pdf]
